# Cross Kingdom Immunity: The Role of Immune Receptors and Downstream Signaling in Animal and Plant Cell Death

**DOI:** 10.3389/fimmu.2020.612452

**Published:** 2021-03-08

**Authors:** Thibault Roudaire, Marie-Claire Héloir, David Wendehenne, Aymeric Zadoroznyj, Laurence Dubrez, Benoit Poinssot

**Affiliations:** ^1^ Agroécologie, Agrosup Dijon, CNRS, INRAE, Univ. Bourgogne, Univ. Bourgogne Franche-Comté, Dijon, France; ^2^ Institut National de la Santé et de la Recherche Médicale (Inserm), LNC UMR1231, Dijon, France; ^3^ LNC UMR1231, Université de Bourgogne Franche-Comté, Dijon, France

**Keywords:** pattern recognition receptors, Toll-like receptors, NOD-like receptors, pathogen-associated molecular patterns, damage-associated molecular patterns, hypersensitive response, regulated cell death

## Abstract

Both plants and animals are endowed with sophisticated innate immune systems to combat microbial attack. In these multicellular eukaryotes, innate immunity implies the presence of cell surface receptors and intracellular receptors able to detect danger signal referred as damage-associated molecular patterns (DAMPs) and pathogen-associated molecular patterns (PAMPs). Membrane-associated pattern recognition receptors (PRRs), such as Toll-like receptors (TLRs), C-type lectin receptors (CLRs), receptor-like kinases (RLKs), and receptor-like proteins (RLPs) are employed by these organisms for sensing different invasion patterns before triggering antimicrobial defenses that can be associated with a form of regulated cell death. Intracellularly, animals nucleotide-binding and oligomerization domain (NOD)-like receptors or plants nucleotide-binding domain (NBD)-containing leucine rich repeats (NLRs) immune receptors likely detect effectors injected into the host cell by the pathogen to hijack the immune signaling cascade. Interestingly, during the co-evolution between the hosts and their invaders, key cross-kingdom cell death-signaling macromolecular NLR-complexes have been selected, such as the inflammasome in mammals and the recently discovered resistosome in plants. In both cases, a regulated cell death located at the site of infection constitutes a very effective mean for blocking the pathogen spread and protecting the whole organism from invasion. This review aims to describe the immune mechanisms in animals and plants, mainly focusing on cell death signaling pathways, in order to highlight recent advances that could be used on one side or the other to identify the missing signaling elements between the perception of the invasion pattern by immune receptors, the induction of defenses or the transmission of danger signals to other cells. Although knowledge of plant immunity is less advanced, these organisms have certain advantages allowing easier identification of signaling events, regulators and executors of cell death, which could then be exploited directly for crop protection purposes or by analogy for medical research.

## Introduction

Eukaryotic cells have evolved complex defense mechanisms in order to combat microbial challenges and preserve organism integrity. Both plants and animals are endowed with a conserved innate immune system able to neutralize pathogens and to contain the infection. It uses specialized receptors to detect microbial patterns, pathogen-derived compounds, and danger signals and elicits an adapted response. The immune response includes a transcriptional reprogramming, the production of antimicrobial molecules, the activation of a regulated cell death program in infected cells and the release of soluble factors such as cytokines and phytohormones able to signal away from the original infection site and alert the host organism of danger. In plants, a systemic resistance is transiently established to prevent forthcoming microbial assault (1). In addition, vertebrates have evolved an adaptive immune system involving specialized cells able to produce a stronger, specific immune response and ensure a long-term protection.

The activation of cell death processes at the site of pathogen attack constitutes an efficient strategy shared by plants and animals to protect the organism from pathogen invasion by directly destroying the pathogen niche. Cell death can also produce alert signals for neighboring cells through the release of intracellular components that can elicit or amplify the anti-microbial response. During a microbial infection, three types of regulated cell death are classically described in animals for confining pathogen progression: apoptosis, pyroptosis, and necroptosis (2, 3). Apoptosis is defined by specific morphological criteria that include cell shrinkage, condensation of the chromatin and fragmentation of the nucleus, plasma membrane blebbing, and the formation and release of apoptotic bodies that are engulfed through a phagocytosis-like process named efferocytosis. At the molecular level, apoptosis involves a cascade of events that culminates in the activation of specific proteases belonging to the caspase family responsible for cell dismantling (2). Of note, apoptosis is a “silent form” of cell death that does not directly cause inflammatory response because of conserved plasma membrane integrity and efferocytosis. Efferocytosis has even been linked to the resolution of inflammation, required for the clearance of dead cells after infection, reducing the production of inflammatory factors by phagocytes and progressively allowing the restoration of homeostasis (4). On the opposite, pyroptosis and necroptosis are associated with the release of pro-inflammatory molecules allowing the establishment of the adaptive immunity. They both involve pore-forming proteins [gasdermin-D (GSDMD) in pyroptosis and mixed lineage kinase domain-like (MLKL) in necroptosis] that trigger membrane permeabilization and osmotic imbalance leading to cell swelling. Many connections exist between signaling pathways leading to apoptosis, pyroptosis, and necroptosis, and these cell death processes can occur simultaneously.

Recognition of invading microbes by plants can also trigger a specific cell death referred to as the hypersensitive response (HR) which efficiently blocks the spreading of biotrophic pathogens in healthy tissues by limiting their access to plant metabolites (5). However, the molecular events involved in HR have not yet been completely deciphered. HR-associated cell death is characterized by an early rupture of the plasma membrane associated with some apoptosis-like features such as cytoplasm shrinkage, chromatin condensation, and nucleus disruption (6). These events are associated with plant-specific cell death features including the dismantling of tonoplast and the vacuolar collapse (7). Consequently, the release of active hydrolases and proteases from collapsed vacuoles can trigger autophagy-like processes (8). Thus, HR appeared as a plant regulated necrosis displaying some feature of necroptosis or pyroptosis in animals (5, 9). As observed during necroptosis and pyroptosis, leakage of the cellular content can constitute alert signals for neighboring cells and prepare them to cope with infections. All of these concerted events insure a global and effective defense response.

The theory that innate immune response is elicited by specialized receptors which recognize conserved microbial components referred to as pathogen-associated molecular patterns (PAMPs) was laid out by Medzhitov and Janeway (10), rewarded by the Nobel Prize in 2011. The first pattern recognition receptor (PRR) was described in plants as a cell surface receptor encoded by the rice gene *Xa21*, which confers resistance to the bacteria *Xanthomonas oryzae* pv. *Oryzae* (11). For many years, two major strategies to study the plant innate immunity have existed. The first was based on a genetic approach: certain varieties of a plant species express *R* genes leading to the perception of microbial effectors encoded by avirulence genes (*Avr*) and then generally to the establishment of the HR. This is the basis of the gene-for-gene concept (*R-Avr*) described by Flor on flax and then widely used for genetic breeding of crop plants (12). The second used a biochemical approach coupled with pharmacological studies. Several teams have purified microbial-derived compounds, commonly referred to as elicitors (chitin, flagellin, elicitin, β-glucans, …) able to trigger plant immune responses. They also characterized high-affinity plasma membrane binding sites interacting with elicitors and showing biochemical features of receptors. To combine the results of these two approaches and to get closer to the concept of PAMPs existing in mammals came the concept of PAMP-Triggered Immunity (PTI), that takes into account the recognition of PAMPs by surface receptors, and Effector-Triggered Immunity (ETI), related to the recognition of effectors (pathogen *Avr* gene products) by intracellular receptors (encoded by *R* genes). However, this PTI-ETI concept proposed by Jones and Dangl (13) was a bit controversial because it was too binary (ETI or PTI) and did not reflect all the existing shade of gray levels of plant immune responses, notably during symbiotic interactions. Recently, new models have emerged in the plant innate immunity with the concept of invasion patterns (14) and signs of danger perceived by a complex plant surveillance system that can produce secondary host-derived immunogenic factors termed phytocytokines (15).

This review aims at highlighting similarities and specificities of the immune responses existing in mammals and plants, mainly focusing on recent advances in the discovery of immune receptors and the involvement of signaling pathways leading to cell death.

## Membrane-Associated PRRs

**Figure 1:**
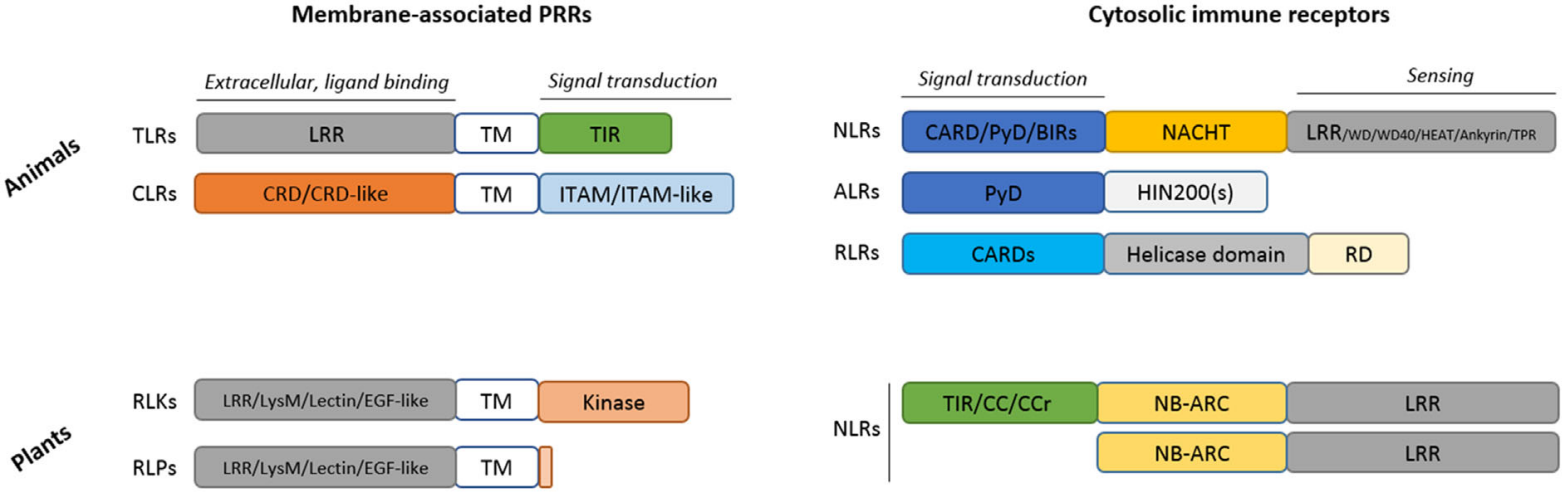
Structural comparison of the main immune receptors found in animals and plants. ALR, AIM2 (absent in melanoma 2)-like receptors; BIR, Baculovirus Inhibitor of apoptosis protein Repeat; CARD, caspase recruitment domain; CC, coiled coil domain; CCr, CC-RPW8; CLR; C-type lectin receptors; CRD, Carbohydrate-Recognition Domain; EGF-like, Epidermal Growth Factor like; HIN200(s), Hematopoietic Interferon-inducible Nuclear protein with a 200 amino acid repeat; ITAM, Immunoreceptor Tyrosine-based Activation Motif; LRR, leucine-rich repeat; LysM, Lysin Motif; NACHT, NAIP (neuronal apoptosis inhibitory protein), CIITA (MHC class II transcription activator), HET-E (incompatibility locus protein from *Podospora anserina*) or TP1 (telomerase-associated protein); NB-ARC, Nucleotide-Binding domain Apaf1, Resistance, CED4; NLR, Nucleotide-binding and oligomerization domain (NOD)-Like Receptor (animals) or Nucleotide-Binding Domain (NBD)-containing LRRs (plants); PYD, Pyrin effector Domain; RD, Regulator Domain; RLK, Receptor-Like Kinase; RLP, Receptor-Like Protein (contain a short cytoplasmic domain devoid of kinase activity); RLR, RIG-I–like receptors; TIR, Toll/Interleukin-1 receptor; TM; Transmembrane; TLRs, Toll-like receptors.

Cells employ a large number of cell surface or endosomal receptors to sense PAMPs and endogenous danger signals referred to as damage-associated molecular patterns (DAMPs) to engage defense responses. In vertebrates, membrane-bound immune receptors belong to the Toll-like receptors (TLRs) family, which are located either at the cell surface or within the endosomal compartment, and C-type lectin receptors (CLRs) located at the cell surface. Plant PRRs are mainly plasma membrane-localized and are divided into two categories: the receptor-like kinases (RLKs) and the receptor-like proteins (RLPs) (**Figure 1**). In both plants and vertebrates, recognition of PAMPs/DAMPs by membrane PRRs primarily activates transcriptional programs that culminate in the production of antimicrobial molecules and in the implementation of an adaptive response of the host to counteract the pathogen attack.

**Figure 2:**
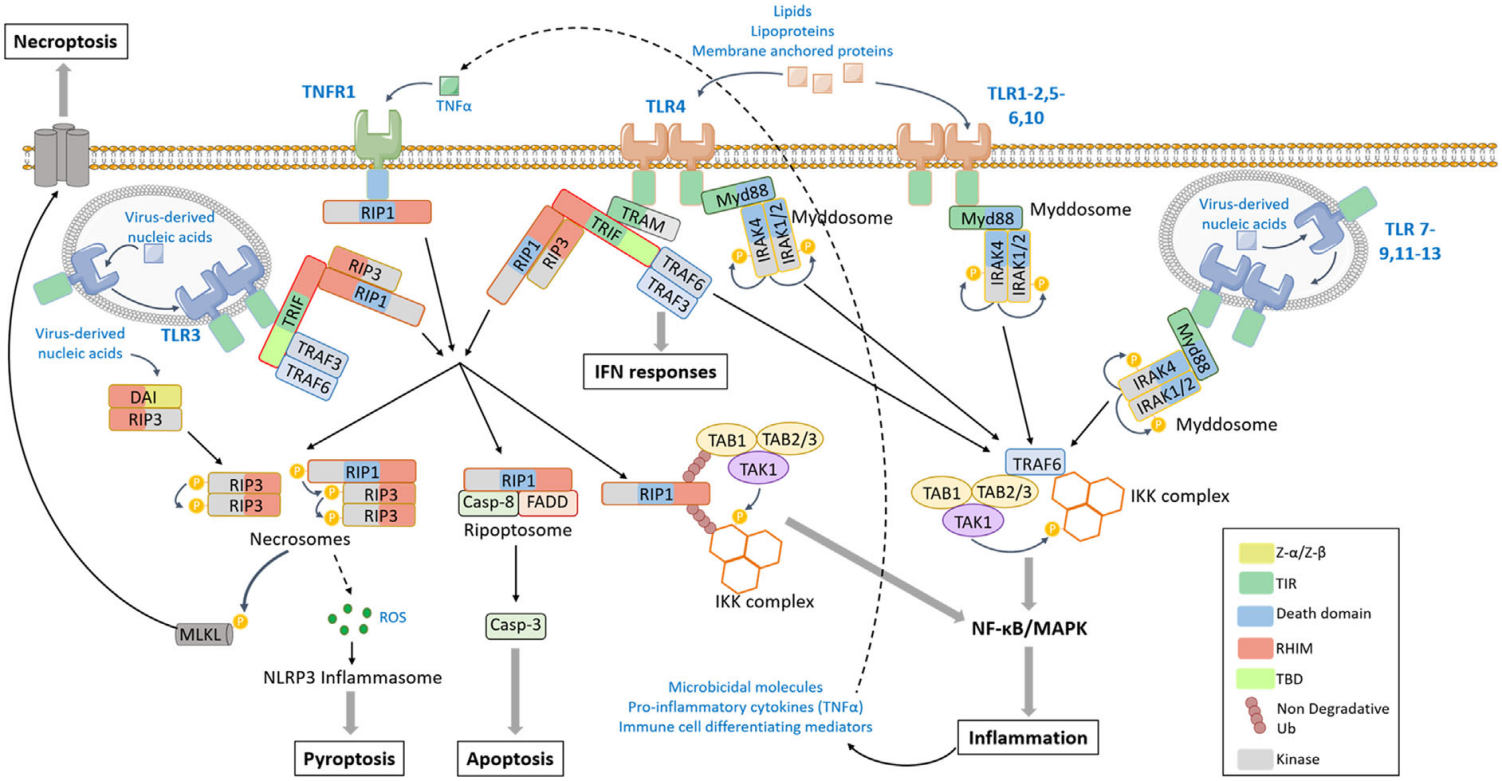
TLR-mediated signaling pathway. The activation of TLR (toll like receptor) 1–2, 4–6, or 10 by lipids, lipoproteins or membrane-anchored proteins and the activation of TLR7-9, 11–13 by virus-derived nucleic acids induce the dimerization of TLRs and the recruitment of Myd88 (myeloid differentiation factor 88) and IRAKs *via* homotypic interaction domains, forming the Myddosome. IRAKs catalyze phosphorylation cascade leading to the recruitment of TRAF6 [tumor necrosis factor (TNF) receptor associated factor 6]. In turn, TRAF6 promotes the activation by proximity of TAK1 (tumor growth factor-β-activated kinase 1)/TAB1-3 (transforming growth factor-activated kinase1-binding protein 1, 2, and 3) and the IKK (Inhibitor of κB kinase) complexes that result in the activation of MAPK (Mitogen-activated protein kinases), NF-κB (nuclear factor-kappa B)-signaling pathways, and pro-inflammatory response. TLR3 and TLR4 stimulation induces the recruitment of TRIF (TIR-domain-containing adaptor-inducing IFN-β) through a TIR-TIR homotypic interaction. The adaptor TRAM (Trif-related adaptor molecule) serves as a bridge between TLR4 and TRIF. In turn TRIF can recruit TRAF3 that engages IFN (Interferon) response, TRAF6 that leads to MAPK and NF-κB activation, or the kinases RIP (Receptor Interacting Protein) 1 and/or RIP3. When poly-ubiquitinated, RIP1 can recruit TAK1/TAB1-3 and IKK complexes and activates the pro-inflammatory response. In a non-ubiquitinated form, RIP1 can assemble with FADD and caspase-8 to form the ripoptosome that leads to caspase cascade activation and apoptotic cell death or can activate RIP3 in the necrosome. RIP3 catalyzes the activating phosphorylation of MLKL, which oligomerizes and translocates into the plasma membrane to form pores and induces necroptosis. The DAI (DNA-dependent activator of IRFs) can also directly recruit RIP3 *via* RHIM homotypic interaction to induce MLKL phosphorylation and necroptosis in response to virus-derived nucleic acids. The necrosome has also the ability to induce ROS (Reactive oxygen species) production resulting in NLRP3 (NOD (Nucleotide-binding oligomerization domain)-like receptor protein) inflammasome and pyroptosis (details of pyroptosis available in **Figure 4**). The TLR-mediated production of TNF-α can induce an autocrine stimulation of TNFR1 that can lead to RIP1 engagement and an amplification of the signal.

Membrane-bound PRRs are composed of an N-terminal extracellular domain that functions as a ligand recognition and binding domain, a transmembrane intermediate and a C-terminal cytoplasmic signal transducing domain (**Figure 1**). In animals, PAMPs are recognized by tandem copies (16–22) of LRR (leucine-rich repeat) in TLRs and by C-type lectin-like domains in CLRs (23). Thirteen TLR paralogs were described in vertebrates. Cell surface TLRs (TLR1, 2, 4–6, and 10) recognize components of microbial membranes such as lipids, lipoproteins, membrane anchored proteins, or extracellular proteins bound to pathogens such as heat shock proteins (HSP) 60 and 70 while TLRs found within endosomal compartment (TLR3, 7–9, and 11–13) likely sense virus and bacteria-derived nucleic acids or endogenous nucleic acids in some pathological conditions. On the other hand, CLRs are dedicated to the defense against fungal infections (23). CLRs can also sense commensal fungi and thus contribute to maintain homeostasis (24, 25). Molecular mechanisms of signal transduction in animal cells are well characterized. They involve the presence of conserved modular domains found in receptors, adaptor proteins or signaling effectors and mediate, through homotypic interaction, the assembly of oligomeric signaling platforms favoring the activation by proximity of signaling effectors. In TLRs, the intracytoplasmic signal transduction domain is the conserved Toll/IL-1 receptor (TIR) domain, also found in the adaptor proteins myeloid differentiation factor 88 (MyD88), MyD88 adaptor-like (MAL), TIR-domain-containing adaptor-inducing IFN-β (TRIF), TRIF-related adaptor molecule (TRAM), and sterile *α*- and armadillo-motif-containing protein (SARM). Ligand binding triggers TLR homo- or hetero-dimerization, conformational change in their intracellular TIR domain and the consecutive recruitment *via* TIR-TIR homotypic interaction of adaptor proteins to form a signaling platform and initiate downstream signaling pathway. TLR stimulation engages MAPKs and/or NF-κB and ultimately promotes inflammatory response that constitutes the first line of defense in mammals and appears critical to maintain homeostasis and tissue integrity. This includes the production of antimicrobial molecules, pro-inflammatory cytokines, and immune cell differentiating mediators responsible for the recruitment and activation of specialized immune cells to the site of infection (**Figure 2**) (16). However, in some situations such as a sustained infection, the presence of pathogens that resist to inflammatory defense or in some pathological conditions [e.g., some cancers, X-linked lymphoproliferative syndrome type 2 (XLP-2)] (17, 18), several TLRs such as TLR3 that recognizes virus-derived double stranded RNAs and RNAs released from damaged cells, and TLR4 that senses LPS from Gram-negative bacteria, can also trigger cell death (19–21). For CLRs, the signal transduction motif is the immunoreceptor tyrosine-based activation motif (ITAM) or ITAM-like motif that mediates the binding and the activation *via* phosphorylation of the spleen tyrosine kinase (Syk). Generally, CLR stimulation does not trigger cell death but results in MAPKs, NF-κB, or interferon-regulatory factors (IRFs) activation (23).

By comparison, plants have evolved hundreds of membrane PRRs able to recognize a wide variety of PAMPs such as flagellin, peptidoglycan, chitin, elicitin, polygalacturonase, and xylanase (22, 26) or DAMPs such as fragments of the plant cell wall extracellular matrix (oligogalacturonides from pectins, cellobiose, and cellodextrines from cellulose and xyloglucans from hemicellulose) (27, 28), extracellular ATP, NAD^+^, or secondary endogenous phytocytokines such as systemin, rapid alkalinization factors (RALFs), pathogen-associated molecular pattern-induced peptides (PIPs), and elicitor peptides (PEPs) (15). The diversity of sensed PAMPs is made possible thanks to the presence of different extracellular domains (**Figure 1**). For example, LRRs found in flagellin sensing 2 (FLS2) sense flagellin or its 22-amino acid epitope flg22 (29, 30), whereas Lysin motif (LysM) specifically recognizes chitin oligosaccharides in chitin elicitor receptor kinase 1 (CERK1) associated with LYK4/5 (31–34) or peptidoglycan with lysin-motif proteins LYM1/LYM3 (35). Epidermal growth factor (EGF)-like domain present in the wall-associated kinase 1 (WAK1) senses oligogalacturonides released during the degradation of the plant cell wall by fungal hydrolases (36, 37), whereas the lectin domains present in AtLecRK1.9 (DORN1) and AtLecRK1.8 perceive extracellular ATP and NAD^+^, respectively (38, 39).

**Figure 3:**
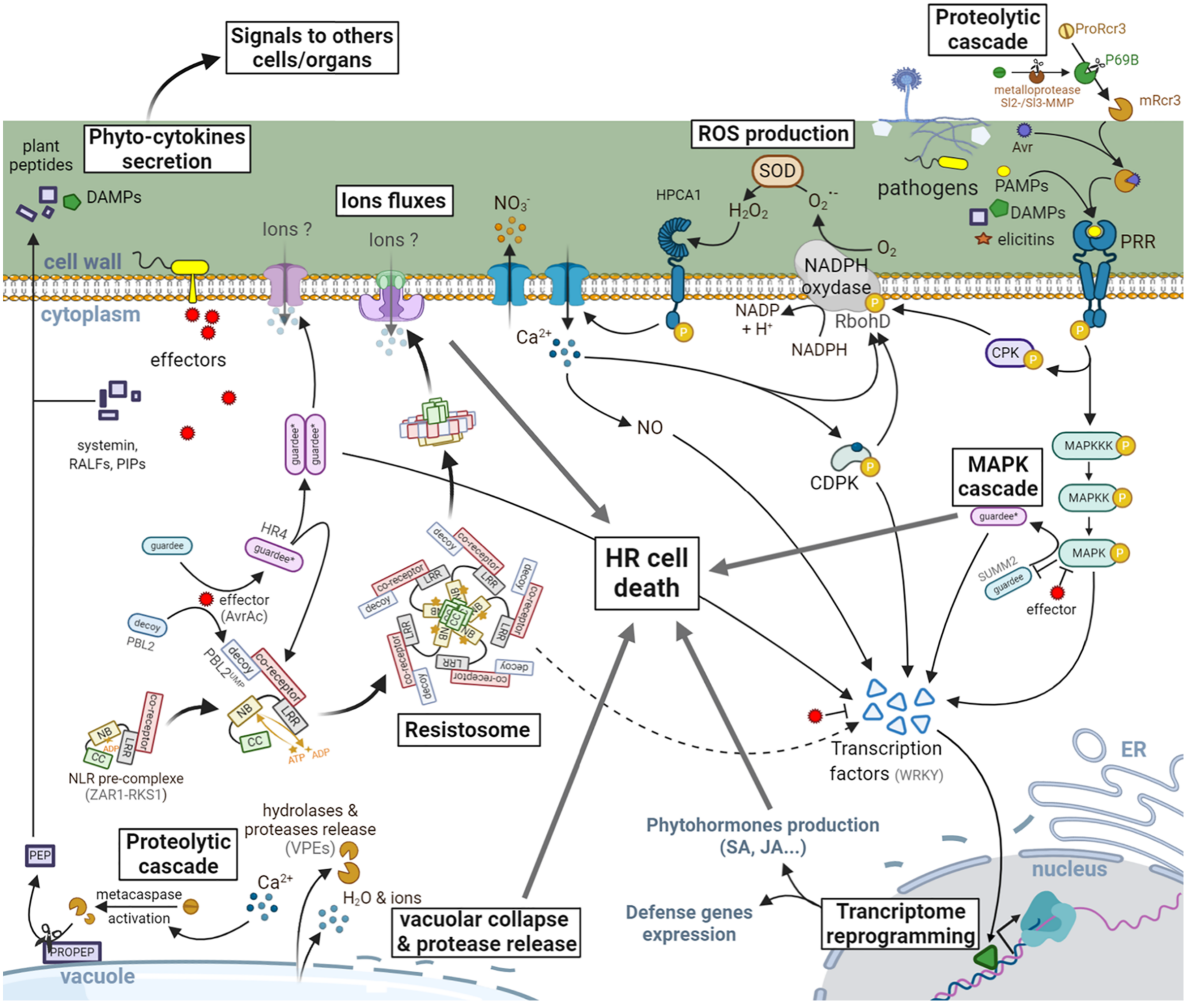
Signaling events leading to HR in plants. PRRs are activated by the recognition of eliciting molecules resulting from the degradation of plant cells (DAMPs) or released by the pathogens (PAMPs, elicitins, apoplastic avirulence factors: Avr). The signal is then transduced by a cascade of phosphorylation events involving MAPKs, cytoplasmic protein kinases (CPKs), and transcription factors, mainly from the WRKY family. This phosphorylation can also activate the NADPH oxidase RbohD, leading to the production of ROS [${\text{O}}_{2}^{.-}$ transformed into hydrogen peroxide (H_2_O_2_) by a superoxide dismutase (SOD)]. An influx of intracellular Ca^2+^, initiated quickly after perception of H_2_O_2_ by HPCA1 leads to the production of nitric oxide (NO), as well as the activation of transcription factors *via* the calcium dependent protein kinases (CDPKs). This is followed by a reprogramming of the transcriptional activity leading to the expression of defense genes involved in the synthesis of phytohormones (SA, JA, …), the antimicrobial phytoalexins or even the release of hydrolytic enzymes (glucanases, chitinases, …) from the pathogenesis-related proteins family. In the meantime, effectors secreted by pathogens to counter the plant’s defenses can also be directly or indirectly (*via* the recognition of a modified host-protein) recognized by NLRs. This recognition generally induces a conformational change in the protein (noted here by an asterisk and a color change), allowing the exchange of ADP by an ATP and therefore the activation of the NLR leading in some cases to macromolecular complexes such as the resistosome or to the activation of transcription factors. These larger-scale molecular complexes have been proposed to act *via* the recruitment of other signaling actors leading to a potentiation of the defenses already in place or by the formation of pores in the plasma membrane. A HR cell death is then observed locally to block the spreading of the pathogen. This will also be associated with the release of DAMPs, phytohormones and phyto-cytokines which will transmit information to neighboring cells and organs to prevent future infections in healthy tissues. Some plant peptides (e.g., PEPs) can be matured by metacaspase-mediated cleavage and released in the apoplast to prime immune responses in neighboring cells, thus enabling the establishment of a local resistance.

Unlike animal PRRs that require the recruitment of adaptor proteins to transduce signal, plant membrane-associated RLKs are endowed with kinase activity thanks to the presence of a cytoplasmic kinase domain (KD) (**Figure 1**). PAMP-sensing induces oligomerization of RLKs and kinase activation that directly triggers numerous cellular responses (**Figure 3**) such as ion fluxes across the plasma membrane, a burst of reactive oxygen species (ROS), a phosphorylation cascade activating MAPKs, and transcription factors leading to the expression of defense genes which encode enzymes involved in the production of defensive secondary metabolites (e.g., phytoalexins, callose, and lignin that reinforce plant cell wall) (26). Because of their very short cytoplasmic domain (about 24 amino acids) devoid of kinase activity, RLPs are not able to transduce signal but function as co-receptors heterodimerized with RLKs that finally elicit a similar cascade of immune signaling events. Most of the PAMPs and DAMPs recognized by plant cell surface PRRs are unable to elicit specific plant cell death. Only some proteinaceous elicitors secreted by pathogens in the apoplast can trigger HR in a plant species dependent manner.

## Nucleotide-Binding Oligomerization Domain (NOD)-Like Receptors (NLRs)

**Figure 4:**
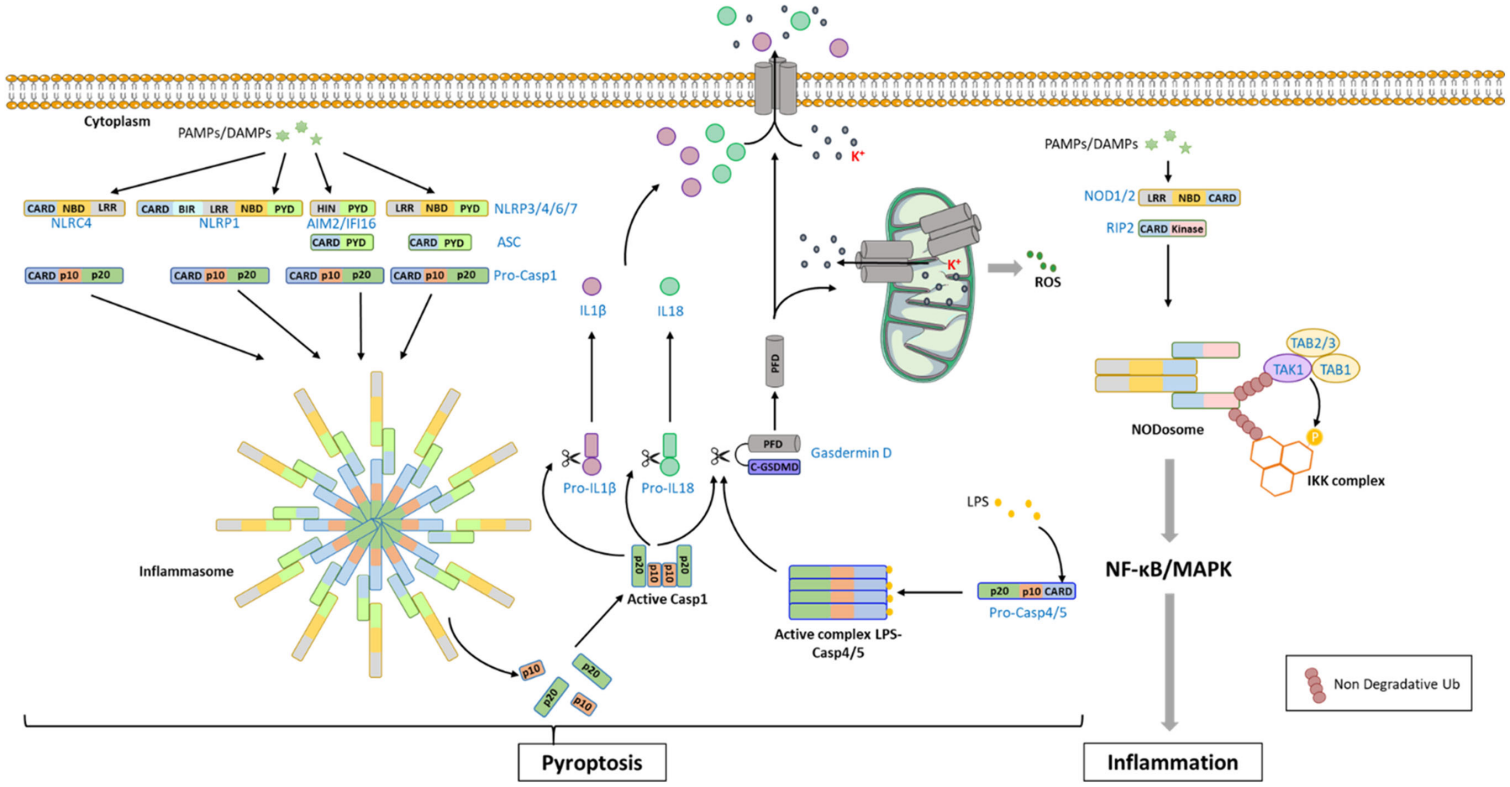
NLR-mediated signaling pathway in mammals. The recognition of PAMPs/DAMPs [Pathogen-Associated Molecular Patterns/Damage-Associated Molecular Patterns) by NLRs (NOD (Nucleotide-binding oligomerization domain)-like receptor proteins: NLRCs, NLRPs] promotes the recruitment of procaspase-1 (Pro-Casp1) through homotypic domain interaction, forming a large cytoplasmic complex named inflammasome. In some situation, the adaptor ASC (apoptosis-associated speck-like protein containing a CARD domain) can take part to the complex, bridging the receptor to procaspase-1. The procaspase-1 undergoes auto-activation and processing of its 2 active sub-units (p10 and p20) which assemble into a tetrameric active form. Active caspase-1 catalyzes the cleavage of proIL-18 (proInterleukine-18), proIL-1β (proInterleukine-1β) into mature cytokines, and gasdermin-D releasing the PFD domain that can oligomerizes and anchors into the membrane to form pores. Processing of gasdermin-D can also be ensured by caspase-4 and caspase-5, which are activated after LPS binding. The gasdermin PFD can also form pores into the mitochondrial membrane that induce K^+^ release and ROS production. In addition, pores created in the plasma membrane by PFD also lead to IL1β and IL18 release and to an ion imbalance that finally results in pyroptosis. The recognition of PAMPs/DAMPs by NOD1/2 promotes the formation of the NODosome composed of the sensor and the protein RIP2 (Receptor Interacting Protein 2) through CARD-dependent homotypic interaction. RIP2 can then recruit TAB1/TAB2/3/TAK1 and IKK complexes that engage MAPK and NF-kB signaling pathways leading to the production of pro-inflammatory cytokines.

Nucleotide-binding oligomerization domain (NOD)-like receptors (NLRs), also generalized as nucleotide-binding domain (NBD)-containing LRRs (NB-LRR in plants), are a class of immune proteins found across both plant and animal kingdoms, with some exceptions in taxa such as Algae, Nematoda, and Drosophila. They were previously classified in plants as R-proteins in the gene-for-gene model. In the animal kingdom, NLRs are the most represented family of cytosolic immune receptors, along with ALRs [AIM2 (absent in melanoma 2)-like receptors] and RLRs (RIG-I–like receptors) (**Figure 1**). ALRs recognize cytosolic DNAs and RLRs sense cytosolic RNAs that include virus nucleic acids as well as endogenous microRNAs (miRNAs) whereas NLRs sense cytosolic PAMPs (e.g., bacteria-secreted toxins, components of bacteria with pore forming activity, internalized LPS, viral proteins) but also markers of intracellular stresses acting as DAMPs such as ATP, uric acids, ROS, metabolic products, or cell-derived peptides that are released by cells in response to endogenous (e.g., ER stress, disruption of ion gradients) or environmental stresses. Stimulation of cytosolic immune receptors can trigger inflammatory response. However, for most of them, the main response is the release of the pro-inflammatory molecules IL-1β and IL-18 and pyroptosis (**Figure 4**). NLR stimulation can also indirectly lead to apoptosis, necroptosis, or activate autophagy process to clear pathogen (40). In plants, NLRs directly or indirectly detect pathogen-secreted effectors (i.e., virulence proteins) to generally promote HR cell death.

Although NLRs are found in both animals and plants, they seem to result from an independent evolutionary process (41). They are characterized by the presence of a central NBD (also named NOD) that may be originated from a prokaryotic class of AAA^+^ ATPases. Both mammals and plants would have selected the NBD for the flexibility of its architecture. It catalyzes the ADP/ATP exchange which promotes the NLR oligomerization and activation. ATP-bound active forms are generally unstable and rapidly recycled into inactive forms or degraded. This mode of activation allows the NLR to quickly react after detection of pathogens and enable to preserve the organism from a costly and useless mobilization of defenses in their absence. Animal NLRs are characterized by a “NACHT” [NAIP (neuronal apoptosis inhibitory protein), CIITA (MHC class II transcription activator), HET-E (incompatibility locus protein from *Podospora anserina*) or TP1 (telomerase-associated protein)] NBD subtype while plants NLRs possess an “ARC” (Apaf1, Resistance, CED4) NBD subtype, also found in some animal adaptor proteins involved in apoptosis such as Apaf-1 (42).

In addition to NBD, LRR is common to both sides. As in animal TLRs, this domain is involved in sensing microbe effectors or modified host-proteins. As an alternative, some animal NLRs own other motif domains with sensing activity such as WD/WD40, HEAT, Ankyrin, or TPR (tetratricopeptide) motifs. LRR domain is widespread among immune receptors of any kinds, probably because of its pre-disposition for forming mismatches thus easily creating diversity and therefore new potential recognition sites, as suggested by Baggs et al. (43). After invasion pattern recognition, LRRs undergo a conformational change allowing NLR switch from an inactive NBD-ADP-bound closed conformation to an active NBD-ATP-bound open conformation and the recruitment of downstream signaling effectors (44, 45). LRR has also the ability to negatively regulate the NBD through intramolecular interactions that prevent its oligomerization (46).

The domains found in the N-term part of the protein are involved in the transduction of downstream signaling (45, 47). In animals, these domains belong to the death-fold superfamily which are homotypic interacting domains also found in adaptor and signaling molecules. The pyrin effector domain (PYD) is the most represented and characterizes the NLRP (NOD-like receptor protein) sub-family of NLRs (also named NALP). The NLRC sub-family contains one or two caspase-activation and recruitment domains (CARD), NLRA the acidic transactivation domain and NLRB the baculoviral inhibitory repeat-like domain (BIR) (**Figure 1**) (40).

In plants, if present, the N-term accessory domain could be of three main types: TIR defining the TIR-NB-LRR (TNLs) group, CC (Coiled-Coil) which defines the CNLs subgroup of NLRs, or CCr (the CC domain subtype with high similarity with the non-NBD-LRR resistance gene RPW8) that characterizes RNLs (48, 49). In addition, different structure variants lacking one or two of the previously described NLR domains can be found (42, 50, 51). Some of these truncated forms, also termed “adapters” or “helpers”, amplify the immune response in plants or could serve as effector baits.

Because of the wide diversity and shorter lifespan of pathogens, different modes of NLR-mediated detection of pathogens have emerged, allowing them to bypass the recognition issue due to their obvious slowly evolution (43, 52). In addition to the classic direct detection of one effector by its related NLR, different ways to activate NLRs have been observed such as the “guard and decoy concept” (42). In this strategy, a decoy protein is in close relation with and constitutively represses the activity of a guard NLR. Effectors or endogenous danger signals mediate decoy modification such as direct cleavage, phosphorylation, acetylation, or uridylation (53–56). These post-translational modifications (PTMs) finally disrupt the inhibition signal allowing the NLR to be fully active (42). In this way, many pathogen effectors can be only detected by monitoring a small number of targets, whether directly involved in defenses (guardee model) or not (decoy model). Moreover, as NLRs are often working by pairs or oligomers, it is not rare to observe an “autoimmune” phenotype, i.e., a constitutive activation of NLRs in the absence of pathogen. This phenotype can result from mutations within the molecule (57, 58) or might also be the consequence of the absence of its related inhibitor, as well as an inappropriate negative interaction between closely related NLRs (59, 60). This is particularly common in the case of crossed plant populations, where inappropriate allelic combinations generate underdeveloped or self-deteriorating seedlings due to the fitness imbalance between growth and defense (61, 62). This phenomenon, also known as hybrid incompatibility, thus facilitates the natural selection of related units. In connection with this, integration of decoy domains inside the NLR protein may have been selected more easily, as the probability of recombination being more important in genetically distant partners than in linked proteins. This then gives rise to the last NLR activation strategy described by Jones et al. (42), the “integrated decoy” model, exemplified by the RRS1 TLR that contains an integrated WRKY transcription factor domain, target of numerous effectors, or the NLR RGA5 that holds an integrated heavy metal-associated copper binding domain.

## Downstream Signaling Events Leading to Cell Death in Animals

In Animals, although many interplays between cell death signaling pathways exist, pyroptosis is primarily the result of the stimulation of intracellular immune receptors (NLRs and cytosolic DNA sensors) while apoptosis and necroptosis are mainly triggered by membrane-associated TLRs.

### NLR-Induced Pyroptosis

The signaling pathway induced by NLR stimulation has been extensively studied in animals and is about to be well characterized [for review, see (63)]. Sensing of PAMPs or DAMPs by the C-term domain triggers NLRs oligomerization and the recruitment of downstream adaptors or signaling proteins to the N-terminal death fold domain *via* homotypic domain interaction, forming large, cytoplasmic, ring-like multi-protein complexes. The best characterized are known as inflammasomes and result in pyropotosis. However, some NLRs such as NOD1 and NOD2 from the NLRC subfamily are not able to induce the assembly of an inflammasome but of a NODosome that ultimately results in the activation of a transcriptional program through NF-κB and/or MAPK signaling pathways (**Figure 4**) (64–66).

Inflammasome is built by the association of a cytoplasmic immune receptor and the effector pro-caspase 1, a cysteine protease from the caspase family (**Figure 4**). Pro-caspase 1 is composed by a CARD-containing prodomain and two active subunits. Its recruitment into the inflammasome *via* CARD-dependent homotypic interactions induces an activating dimerization and subsequent auto-cleavage of the two active subunits that assemble into the tetramer active form. Once activated, caspase-1 induces the processing of the pro–IL-1β and pro–IL-18 into mature cytokines. Caspase-1 can also catalyze the cleavage and activation of the pore-forming protein gasdermin D. Gasdermin D belongs to the pore-forming protein family gasdermin (GSDM) characterized by the presence of an N-terminal pore-forming domain (PFD). It is synthetized as a precursor composed of two domains linked by a loop. Caspase-1 cleaves Gasdermin D within the loop, releasing the PFD that oligomerizes and anchors into the inner plasma membrane to form pores. Inflammasome-mediated Gasdermin D activation results in IL-1β and IL-18 release, and ionic imbalance that culminates in cell swelling (67, 68). The cleavage of gasdermin D can also be performed by the human CARD-containing caspase-4 and -5 (mouse caspase-11) that is activated in the so-called non-canonical inflammasome thanks to their ability to directly sense LPS by their CARD domain (69). Interestingly, Gasdermin D is also able to form pores into bacterial and organelle membrane such as mitochondria or endoplasmic reticulum that can result in potassium efflux, calcium mobilization and ROS generation (70).

Among NLRs, NLRP1, -3, -4, -6, -7, and NLRC4 can form inflammasome. Moreover, the cytosolic DNA sensors AIM2 and IFI16 also have the ability to form it (71–73). For NLRP3 and AIM2 that do not contain a CARD domain, the molecular adaptor ASC (apoptosis-associated speck-like protein containing a CARD domain), that owns both a pyrin and a CARD domain, is recruited as an intermediate bridge to link the sensor to the pro–caspase-1 (**Figure 4**). ASC adaptor is also observed in the NLRC4 and NLRP1 inflammasomes, stabilizing the interaction between the NLR and the pro–caspase-1 (74). The activation process of NLRP3-inflammasome is the most documented (75). It involves a priming step that can be provided by sensing LPS by TLRs which induces NF-kB-dependent expression of NLRP3 and ASC (76). As the intracellular amount of these proteins have reached a sufficient level, NLPR3-inflammasome activation can be completed by sensing DAMPs (such as ATP) or some PAMPs (viral RNA or proteins, bacterial-derived compounds). Some PTMs such as phosphorylation (77) and de-ubiquitination (78) could also be of importance for the priming step.

### TLR-Mediated Apoptosis and Necroptosis

As described above, TLR-mediated signal transduction involves the assembly of multiprotein signaling platforms thanks to the presence of homotypic interacting domains in the receptors, adaptors and effector proteins (16). Ligand binding triggers TLR homo- or heretodimerization, conformational change in their intracellular TIR domain and the consecutive recruitment *via* TIR-TIR homotypic interaction of adaptor proteins. Schematically, TLR signaling pathways are subdivided into MyD88-dependent and TRIF-dependent signaling. Briefly, MyD88 can bind most of endosomal and cell-surface-TLRs. In turn, it promotes the recruitment of serine/threonine IL-1R–associated kinase (IRAK) family members, *via* homotypic interaction with its C-term death domain (DD), to form a multiprotein complex named Myddosome. IRAK4 catalyzes a phosphorylation reaction that leads to the recruitment of the E3-ubiquitine ligase TRAF6 [tumor necrosis factor receptor (TNFR)-associated factor 6], the subsequent recruitment and activation of TAK1/TAB1/3 and IKK complexes. This signaling platform engages MAPKs and NF-κBs, and that ultimately leads to the expression of pro-inflammatory cytokines that include TNFα (tumor necrosis factor-α) (16) (**Figure 2**).

The endosomal TLR3 and the cell surface TLR4 recruit the adaptor TRIF. While TLR3-TRIF binding is direct *via* TIM-mediated homotypic interaction, TLR4 requires the adaptor TRAM that bridges TLR4 to TRIF. TRIF can recruit TRAF3 and/or TRAF6 thanks to the presence of TRAF-binding motifs in the N-terminal part of the protein. TRAF6 promotes subsequent MAPK or NF-κB-dependent transcriptional program while TRAF3 engages the IRF3-activating signaling pathway and the expression of type I IFN genes (16) (**Figure 2**). In addition to the TIR and TRAF-binding domains, TRIF owns a C-terminal RIP homotypic interaction motif (RHIM) also found in the receptor-interacting kinases (RIPs) RIP1 and RIP3 (79).

The serine/threonine kinase RIP1 has been identified in 1995 because of its capacity of binding death receptors from TNFR superfamily (80). Thus, RIP1-dependent signaling pathway can be activated by TLR3 and TLR4 as described above and also indirectly by the other TLRs *via* the NF-κB-dependent production of TNFα and autocrine stimulation of TNFR1. RIP1 appears as the core component of signaling platforms, at the crossroad between inflammatory response, apoptotic and necroptotic cell death signaling pathways [for review see (81)] (**Figure 2**). When modified by non-degradative ubiquitin chains, RIP1 constitutes a scaffold for the recruitment of the kinase complexes TAK1/TAB1/TAB2 and IKK that activate MAPK and NF-κB-dependent transcriptional response. In a non-ubiquitinated form, RIP1 promotes the assembly of a secondary cytoplasmic cell death signaling platforms thanks to its kinase activity. Associated with the adaptor FADD (Fas-associated protein with DD) and caspase-8, it promotes caspase-dependent apoptotic cell death *via* the activation of the proteolytic cascade involving the caspase-3 (82). RIP1 can recruit its closely related protein RIP3 *via* their respective RHIM domain and forms a necrosome (**Figure 2**). Independently of RIP1, RIP3 can also be directly engaged by TLR3 and TLR4 *via* a RHIM-dependent binding to TRIF, and also by the cytosolic DNA sensor DAI from RLR family that owns a RHIM domain (83).

RIP3 is the main effector of necroptosis (84). It is activated by homodimerization and autophosphorylation. RIP3 promotes the activating phosphorylation of MLKL (85). Once activated, MLKL oligomerizes and translocates to the plasma membrane where it interacts with phosphatidylinositols and forms pores or cation channels responsible for membrane permeabilization and disruption and cell death (84, 86). Necroptosis has been associated with the production of ROS. Deletion of RIP3 completely blocked ROS production suggesting that this event occurs downstream of necrosome activation (87).

The concept of necroptosis has emerged over the two last decades but its contribution to the control of pathogen infections is still not very well understood. Experimental necroptosis model usually requires an inhibition of apoptotic signaling pathway, suggesting that necroptosis occurred as a secondary event (88). The role of RIPK3-mediated cell death in antiviral response was highlighted by the analysis of mice deficient in RIPK3 that appeared highly susceptible to Influenza A virus or vaccinia virus (87, 89). In the same manner, macrophages or lung epithelial cell death induced by some bacteria (that include *Salmonella typhimurium*, *Staphylococcus aureus*, *Staphylococcus marcescens*, or *Streptococcus pneumoniae*) are inhibited by RIPK3 deficiency or necroptosis inhibitors (90–92). Necroptosis could constitute an important defense mechanism against infection with pathogens able to bypass apoptosis induction by expressing anti-apoptotic proteins.

## Downstream Signaling Events Leading to Cell Death in Plants

### NLR-Mediated HR

ETI-related HR cell death is frequently associated with early signaling events such as NLR oligomerization, activation of a MAPK cascade, ROS generation, transcriptional reprogramming, and a later accumulation of phytohormones associated to plant immune responses (93, 94). Once the pathogen is detected through the direct recognition of an effector or *via* a modification of a host molecular target, larger immune receptor complexes are built. Recently, an NLR supramolecular structure termed resistosome and showing similarities with the mammalian inflammasomes has been discovered in plants. In this case, the bacterial pathogen *Xanthomonas campestris* injects into the host plant cells the AvrAC effector which uridylates the PBS1-like protein 2 (PBL2) decoy receptor-like cytoplasmic kinase (RLCK). This modification (PBL2^UMP^) triggers its association with the second RLCK resistance-related kinase 1 (RKS1) that interacts with the NLR Hop-Z–activated resistance 1 (ZAR1). This allows the NBD to become active by exchanging its ADP to ATP (95, 96). Subsequent ATP binding triggers the pentamerization of the ZAR1-RKS1-PBL2 resistosome complex (**Figure 3**). The assembled supramolecular complex forms a funnel-shaped structure with a pore diameter ranging from ~10 to 30 Å. Because of its structural resemblance with the hemolytic pore forming protein fragaceatoxin C and the partial requirement of its association with the plasma membrane for the induction of cell death, Wang and collaborators hypothesized this complex to be pore-forming. The ZAR1 resistosome would thus disrupt the plasma membrane integrity and/or alter the ions homeostasis by acting in a similar manner to the MLKL and gasdermins in mammals (9). Nonetheless, we cannot rule out that the ZAR1 resistosome could also serve as a docking site for others immune actors. Furthermore, some NLRs complexes appear to directly activate the expression of transcription factors, rather than associating with the membrane, to finely tune cell death and immune responses in plants. In that way, the *Arabidopsis thaliana* (Arabidopsis) helper NLR AtNRG1 (N-requirement gene 1) forms a cell death signaling hub when it is associated with the heterodimer formed by the lipase-like protein enhanced disease susceptibility1 (AtEDS1) interacting with the senescence-associated gene101 (AtSAG101) (97–99). However, if AtEDS1 interacts with phytoalexin-deficient 4 (AtPAD4), this molecular complex associates with another helper NLR AtADR1 (accelerated disease resistance 1) to promote the expression of immune genes *via* transcriptional reprogramming (99).

In Arabidopsis, HR cell death pathway is regulated by a MAPK cascade, involving the MAPK kinase kinase MEKK1, the MAPK kinases MKK1/2, and the MAPK MPK4. Interestingly, the CC-NLR protein SUMM2 (suppressor of *mkk1/2*, 2) has been shown to monitor this MAPK cascade (100). Indeed, the Arabidopsis *mekk1*, *mkk1/2*, and *mpk4* mutants or plants in which *MEKK1* has been silenced exhibited spontaneous cell death and constitutive immune responses such as defense gene activation and ROS production (101–106), whereas mutations in SUMM2 suppressed the cell death of *mekk1*, *mkk1/2*, and *mpk4* mutants (100). A screening of Arabidopsis T-DNA insertion lines identified SUMM2, MEKK2, and calmodulin-binding receptor-like cytoplasmic kinase 3 (CRCK3) as key regulators of MEKK1 depletion-induced cell death (107). At the opposite, overexpression of CRCK3 induced a SUMM2- and MEKK2-dependent cell death (106). Altogether, these results suggest that a dedicated plant MAPK cascade plays an important role in the HR signaling, which is under the tight control of different regulator proteins.

The onset of HR is also often associated with the production of ROS. Most of the apoplastic ROS generated during plant-pathogen interactions are produced *via* plasma membrane-localized enzymes with homology to the mammalian phagocytes NADPH oxidases (NOXs) and called respiratory burst oxidase homologs (RBOHs) in plants (108). These enzymes generate apoplastic superoxide ions (${\text{O}}_{2}^{.-}$) that rapidly dismutate to hydrogen peroxide (H_2_O_2_), a well-known microbicide. Following infection by a bacterial pathogen possessing the avirulence gene *AvrRpm1*, HR is reduced in Arabidopsis *AtrbohD* mutant and *AtrbohD/F* double mutant plants, indicating that RBOHs and ROS promote and/or mediate cell death (109). This process requires the concomitant production of nitric oxide (NO; see below). Nevertheless, infection with an avirulent oomycete pathogen in Arabidopsis *AtrbohD/F* double mutant plants caused more HR and resistant phenotype even though ROS production was suppressed (109). Similarly, Arabidopsis *nca1* (no catalase activity 1) and *cat2* (catalase 2) mutants, supposed to have an increased H_2_O_2_ level, surprisingly showed reduced cell death when infected by a bacterial pathogen expressing *AvrRpm1* (110). At the molecular level, the discovery that RBOHD activity is positively and negatively regulated by many PTMs *via* different phosphorylation or ubiquitination events suggests a finely tuned control of the spatio-temporal ROS production during plant-pathogen interaction (111–114). Thus, the connection between ROS and HR still needs clarifications. Interestingly the Arabidopsis LRR-RK HCPA1 has been recently shown to perceive extracellular H_2_O_2_ *via* cysteine oxidation to trigger Ca^2+^ influx, which then leads to immune responses such as stomatal closure to delay the pathogen penetration through this natural opening in plants (115).

### PRR-Triggered Signaling Events Leading to HR

Apoplastic elicitors which trigger HR are more the exception than the rule. Nevertheless, Avr2/4/5/9 proteins secreted by the fungus *Cladosporium fulvum* induce HR in tomato expressing the corresponding surface RLP Cf-2, Cf-4, Cf-5, or Cf-9, respectively (116–118). Similarly, fungal endopolygalacturonases secreted by *Botrytis cinerea* trigger plant cell death in a specific Arabidopsis ecotype (Columbia-0) co-expressing the RLPs RBPG1 (responsiveness to *Botrytis cinerea* polygalacturonases1) associated with SOBIR1 (suppressor of BIR1-1) (119). Elicitin proteins secreted by different species of *Phytophthora* can also trigger HR in different solanaceous plants after recognition by the RLP ELR (elicitin response) which forms a molecular receptor complex with the RLK BAK1 (brassinosteroid insensitive 1-associated receptor kinase 1) (120).

Cryptogein produced by *Phytophthora cryptogea* proved to be an efficient biological tool to study the mechanism underlying HR. More precisely, *Phytophthora cryptogea* induces an immune response in tobacco plants characterized by a HR and a transient systemic resistance conferring protection against numerous micro-organisms, including virulent ones (121). Cryptogein secreted by this oomycete is the major inducer of plant immunity and mimics the effects of the oomycete once applied to tobacco plants and cell suspensions (122, 123). Cryptogein is an elicitin protein of 10 kDa that acts as sterol carriers and could supply *Phytophthora* species with sterols, these latter being sterol auxotrophs (124). *In vitro* binding assays provided first evidences that cryptogein is recognized by a putative plasma membrane receptor (125). Even if its molecular identity has not been reported so far, it is plausible to assume that the RLP ELR fulfils this role (120).

The cellular and molecular processes leading to the cryptogein-induced HR have been widely studied, mostly using tobacco cell suspensions. Cells undergoing cell death show a vacuole shrinkage within few hours (126). This vacuole volume loss has been functionally linked to fast and ample nitrate effluxes across the plasma membrane resulting from the activity of anion-permeable channels (126, 127). Accordingly, inhibition of these effluxes, as well as Ca^2+^ influxes from the extracellular space, suppressed or delayed cell death (128). A similar result was observed in cryptogein-treated tobacco cells in which the activity of protein kinases, including MAPK, has been suppressed (129). All together, these data highlighted that the machinery leading to cell death is a dynamic process requiring early signaling events. These latter also include the production of NO and ROS. More precisely, cryptogein triggers within minutes the activation of a plasma membrane NADPH oxidase (NtRBOHD) which produces ${\text{O}}_{2}^{.-}$ simultaneously dismutated into H_2_O_2_ through the activity of superoxyde dismutases (130). Interestingly, a coordinated action of ROS and NO has been highlighted (131). Indeed, the production of H_2_O_2_ is a prerequisite for NO synthesis and functions as impairment of NtRBOHD expression compromises NO production as well as its involvement in cell death. In turn, NO negatively regulates the level of H_2_O_2_ through the formation of peroxynitrite (ONOO^–^) resulting from the chemical combination between NO and ${\text{O}}_{2}^{.-}$. The formation of ONOO^–^ might mitigate the effects of H_2_O_2_ and provide a mean to control the intensity of cell death. The possibility that NO also mitigates H_2_O_2_ production and the amplitude of HR through the inhibition of NtRBOHD by S-nitrosation, a NO-dependent post-translational protein modification, has been reported in other plant-pathogens models (132). However, the occurrence of this mechanism in cryptogein-treated cells has not been confirmed (131).

Caspase-like activities have also been detected in plants during HR. If genes encoding true orthologs of caspases (cysteine dependent aspartate-directed proteases) are absent in the plant genomes, many proteases involved in HR have been identified such as metacaspases in the cytosol, vacuolar processing enzymes (VPEs) in the vacuole or saspase, cathepsin B or papain-like cysteine protease (PLCP-like Rcr3 or Pip1) in the apoplast (9). Interestingly, plant metacaspases are lysine- and arginine-specific, whereas caspases found in mammals are aspartate-specific, indicating a different substrate specificity of these plant enzymes. Based on their protein structure, the phylogenetic analysis of plant metacaspases indicated three major clades that can be divided into type I with, or without, a zinc finger motif in the N-terminus region, and type II harboring a linker region between the two subunits of 10 and 20 kDa of the caspase-like regulatory and catalytic domains (133). In Arabidopsis, the type I metacaspase AtMC1 promotes HR during biotic stresses whereas AtMC2 acts antagonistically by inhibiting plant cell death without any different pathogen dissemination in both *atmc1* and *atmc2* mutants (134). The type II metacaspase AtMC4 is a calcium-dependent cysteine protease which cleaves the PROPEP1 phyto-cytokine in order to release PEP1 in the apoplast, itself detected by the PEPR1 PRR in neighboring cells to amplify plant immune responses during the damage-triggered immunity (135). The recent resolution of the AtMC4 crystal structure highlights the inhibitory role of the large linker domain which blocks activation and substrate access to the catalytic domain (136). Concerning VPEs, they have been shown to exhibit similar enzymatic properties as the animal caspase 1 except that they are active in the vacuole (7). They possess an autocatalytic conversion of the inactive proprotein (pVPE) into the mature active mVPE (137). In tobacco plants infected by the tobacco mosaic virus (TMV), caspase-1 inhibitors or *VPE* gene silencing reduces the caspase-1–like activity associated to the rupture of the vacuolar membrane normally leading to the virus-induced HR (138). During plant immunity, bacterial harpin-induced cell death was compromised in *Nicotiana benthamiana VPE*-silenced plants (139) and inhibitors of caspase-1 delayed the HR cell death normally triggered in tobacco cells by the oomycete elicitor cryptogein (126). In Arabidopsis, a *vpe* null mutant lacking the four *VPE* genes (*α−*, *β−*, *γ−*, and *δ−vpe*) was unable to show any VPE or caspase-like 1 activity (140) and γ-*vpe* mutant was more susceptible to viral, bacterial or fungal infection (141). The extracellular proteases Pip1 and Rcr3 also actively participate to the perception of the fungal pathogen *C. fulvum* in tomato. *C. fulvum* secretes Avr2 into the apoplast which associates with Rcr3 and Pip1. The mentioned complexes perceived by Cf-2, a RLP, trigger HR and induce resistance to *C. fulvum* (142). Recently, the first proteolytic cascade has been discovered in plants where the extracellular immune protease proRcr3 is cleaved by the subtilase P69B in mature Rcr3 that interacts with Avr2 before that the molecular complex binds to the Cf-2 RLP (143). Interestingly, this P69B subtilase has been previously shown to be itself the target of the matrix metalloproteinases Sl2-MMP and Sl3-MMP (144, 145), suggesting that these MMP are initiator proteases whereas Rcr3 plays an effector role in this cascade, as shown for intracellular caspases in mammals.

## Cell Death Regulations in Mammals and Plants

In host-pathogen interactions, the control of the regulated cell death is crucial for both partners. Host have to adapt their defense responses to contain pathogen development while avoiding their own lethal outcome. At the opposite, pathogens need to modulate the regulated cell death to ensure their infection cycle.

### Cross Regulation Mechanisms Between Immune Signaling Pathways

Organisms need to adapt their response as the infection evolves. Thus, many interplays and cross-regulation mechanisms exist between the different immune cell signaling pathways. The stimulation of one receptor can cause different responses including the activation of transcriptional programs or the engagement of cell death pathways, depending on the nature, the duration and the intensity of the stimuli. These responses can occur simultaneously or successively and cells have the ability to change the response very quickly. In animals, the interplay between TLR and NLR signaling pathways is illustrated by the activation of NLRP3-inflammasome in a two-step process. The first priming step involves the TLR-mediated, NF-κB-dependent expression of *NLRP3*. Then DAMPs or PAMPs can stimulate the NLRP3-inflammasome assembly and pyroptosis (76). Thus, NLRP3-inflammasome-mediated pyroptosis is only activated when the TLR-mediated immune response is not sufficient to neutralize pathogens. As illustrated **Figure 2**, TLR4 is able to trigger NF-kB-dependent pro-inflammatory response, IFN-response, necroptosis, or apoptosis. This implies the presence of accurate regulation mechanisms. In addition to promote the production of microbicidal molecules and pro-inflammatory mediators, TLR-mediated NF-κB activation induces the expression of several survival proteins such as cellular inhibitors of apoptosis (cIAPs) (146). cIAPs act as E3-ubiquitin ligases promoting poly-ubiquitination of RIP1. Therefore, they are required for the TLR-dependent activation of transcriptional programs while they inhibit RIP-dependent apoptotic and necroptotic pathways (18, 147). The presence of a second signal that neutralizes cIAP activity could convert the transcriptional signal to a cell death signal. It is interesting to note that some viruses such as baculovirus express proteins from IAP family (148) that block cell death of infected cells and allow viral propagation. The different cell death signaling pathways are also interconnected and have the ability to regulate each other [for review, see (75)]. Blocking apoptosis signaling pathway is generally a prerequisite for detecting necroptosis because the apoptotic caspase-8 can inhibit the activity of key components of the necroptosis signaling pathway including RIP1 and RIP3 (84, 88). On the contrary, caspase-8 can activate the NLRP3-inflammasome (149) or directly cleave the caspase-1 leading to pyroptosis (150). Conversely, inflammasomes can connect caspase-8 to induce apoptosis (151). The necroptotic effector RIP3 is also able to cause NLRP3-inflammasome activation and pyroptosis *via* ROS production (152, 153).

Although less documented, such cross-regulations between immune receptor-mediated signaling pathways probably exist in plants. Mutations or overexpression of some plant immune receptors such as the RLK CERK1, the co-receptor BAK1, its closest homolog BKK1 (BAK1-like 1) or the RLP BIR2 (BAK1-interacting RLK 2) can trigger an enhanced cell death (154–157). This discovery suggests that a plant surveying system probably guards these immune receptors from inhibition by pathogen effectors to trigger HR (9, 158, 159).

### Modulation of Immune Signaling Pathways by Post-Translational Modifications

Molecular mechanisms that dictate the response to immune receptor stimulation are not completely understood and are subject of intense research. Because of their flexibility and speed of implementation, PTMs constitute a remarkable and effective process to modulate the intracellular signaling and to regulate the communication networks between cell transduction pathways.

In mammals, as reported above, the priming step required for the full activation of NLRP3-inflammasome has also been shown to involve some PTMs of NLRP3 such as phosphorylation (77) and de-ubiquitination (78). A nice example is given by RIP proteins (RIP1, RIP2, and RIP3) for which the recruitment into various multiprotein signaling platforms, the kinase activity and the ability to engage downstream signaling pathways is orchestrated by PTMs, mainly ubiquitination and phosphorylation (160). The serine/threonine kinase RIP1 is recruited to the TLR3 and TLR4-associated signaling complex (**Figure 2**) and RIP2 is associated with the cytosolic NLRs NOD1 and NOD2 (161) (**Figure 4**). When polyubiquitinated RIP1 and -2 function as a molecular scaffold. It promotes the recruitment and the activation of the kinase complexes IKK and TAB1/TAB2/TAK1 that promote a pro-survival and pro-inflammatory response (**Figure 2**) (146, 160, 161). On the other hand the non-degradative ubiquitination also completely inhibits RIP1 kinase activity that is essential for the assembly of secondary cytoplasmic cell death signaling platforms leading to apoptosis or necroptosis (18, 160). Of note, some viruses have developed strategies to counteract the death of infected cells by modulating RIP ubiquitination (162).

The plant immune responses are also finely regulated by PTMs of signaling proteins. The ubiquitin-proteasome system (UPS) plays an essential role in plant immunity. Among UPS components, E3 ubiquitin ligases have been particularly studied. Wang et al. (163) showed that the E3 ligase OsPUB15 interacts with the homodimerized PID2K, a transmembrane RLK, which confers rice resistance against *Magnaporthe oryzae*. Interestingly, the authors demonstrated that the overexpression of *OsPUB15* led to an enhanced resistance against the pathogen, correlated with up-regulation of some defense genes, excessive accumulation of ROS and plant cell death, suggesting that ubiquitination of PID2K could favor its activity. However, in this case, the observed lesions were so important that they conducted to the plant death. Another example of the importance of UPS in plant immunity is provided by studies investigating the function of the ATPase cell division cycle 48 (CDC48). CDC48 is a highly conserved chaperone-like protein from yeast to plants and animals [also named VCP (vasolin-containing protein) or p97 in mammals]. This protein catalyzes the disassembly of protein complexes and/or the extraction of ubiquitinated proteins from membranes or chromatin in order to deliver them to the proteasome. Therefore, it plays an important function in UPS and, more generally, proteostasis (164). Investigations of CDC48 function in plant immunity demonstrated that the tobacco CDC48 isoform rapidly accumulates in its hexameric active structure in tobacco cells exposed to cryptogein (165). A screening for its binding partners allowed to the identification of key regulators of the redox status, including cytosolic ascorbate peroxidase (cAPX) a pivotal enzyme for ROS removal (165, 166). In CDC48-overexpressing tobacco cells, the activity of cAPX was impaired, leading to severe decrease in the cell capacity to respond to oxidative stress (167). Accordingly, a faster and pronounced cell death was observed in those cells. Although speculative, the involvement of CDC48 in cell death could also be explained by its ability to promote the degradation of cell death repressors. In animals, several studies have reported a role for its ortholog p97 in viral spreading (poliovirus, herpes simplex virus, cytomegalovirus, or influenza virus) by regulating the cycle of viral replication in infected cells (168). However, the mechanisms and the relationship with immune receptor-induced cell death have not been clearly demonstrated.

Histone deacetylases (HDACs) of type 2 (HD2s) have also been identified as important regulators of the cryptogein-induced HR in tobacco. HD2s design a plant specific family of nuclear histone deacetylases (169). Two HD2s tobacco isoforms, NtHD2a and NtHD2b, were shown to undergo a fast phosphorylation in tobacco cells treated with cryptogein (170). This process was followed by a decrease both at the transcript and protein levels. Interestingly, silencing of *HD2* in cell suspensions or *in planta* led to a faster and amplified cell death manifested by exacerbated HR symptoms. Based on these data, it has been proposed that NtHD2a and NtHD2b act as constitutive negative regulators of HR by modulating the expression or activity of HR regulators or effectors which identities remain to be discovered. In a similar manner, a role for histone deacetylases in the acquisition of cell resistance phenotype has also been observed in mammal macrophages. An upregulation of HDAC8 has been correlated with the acquisition of a resistance phenotype to anthrax lethal toxin (LeTx). HDAC inhibitors sensitized cells to LeTx-induced pyroptosis while inversely upregulation of HDAC8 prevents LeTx-induced cell death (171).

### Regulation of Immune Signaling Pathways by miRNAs

As NLRs are often tightly linked to strong immune process including cell death, these proteins need to be finely tuned to avoid any deleterious impact on the plant fitness in the absence of pathogen. In accordance, animals and plants possess several regulation processes among which are miRNAs. These regulatory elements function similarly in animals and plants and are in the same way excised from long primary miRNA transcripts by Dicer or Dicer-like enzymes (such as DCL1) before being loaded into an RNA-induced silencing complex (RISC) and to repress the gene by DNA methylation or by cleavage, destabilization or translational inhibition of its messenger RNA (mRNA) (172). These miRNAs are involved in the regulation of different biological processes, and particularly studied in plants in development and defense contexts. In addition to interact with exogenous nucleic acid and defend plant cells against viral pathogens (173), some host miRNAs also target their own transcripts encoding immune receptors such as the NLR proteins (174–177). This is believed to allow them to control their immune reactions in the absence of pathogen and therefore to avoid any unnecessary waste of energy. In addition, since some bacterial and viral pathogens infect their host by blocking its miRNA interference process, the decrease in these miRNAs would also, at the same time, lead to an accumulation of the previously repressed immune receptors, ultimately leading to a potentiation of defenses (174, 176, 178).

### Regulation of Plant Immunity by Phytohormones

Plant immunity is also regulated by a complex network of phytohormones, which integrate signals from biotic and abiotic stresses in order to finely tune the spatio-temporal expression of the different immune responses. Among them, salicylic acid (SA) and jasmonic acid (JA) play major roles and their antagonism is believed to specifically adapt the plant immunity to biotrophic or necrotrophic pathogens, respectively (179). SA has been shown to positively regulate the HR cell death during interaction with biotrophic pathogens whereas JA seems to be more important for the plant resistance against invading necrotrophs or insects. Actually, low level of SA downregulates HR cell death whereas high level of SA triggers plant cell death (180). Moreover, this hormonal balance between SA and JA seems to finely regulate plant cell death locally as SA accumulates into the HR-related cell death zone whereas JA level increases in the surrounding area to act antagonistically with the SA-pathway (181).

### Pathogens Interfere With Cell Death Signaling Pathways to Their Own Benefits

According to their infection cycle, some pathogens also interfere with regulated cell death signaling pathways to their own benefits (182). So, it is generally accepted that viruses and biotrophic pathogens whose survival is fully dependent on the intracellular machinery of host cells can delay or inhibit cell death contrary to necrotrophic ones which take nutrients from dead cells. However, the classification does not always reflect the complexity of the pathogen cycle infection. The strategies used by pathogens to evade host defenses in order to favor their multiplication and spread have been widely studied in animal cells. Many viruses or bacteria deliver anti-apoptotic proteins that directly block apoptotic, necroptotic and/or pyropotic machineries. For example, caspase-1 and/or caspase-8 involved in pyroptosis and apoptosis (**Figures 2** and **4**), respectively, can be directly inhibited by serpin (serine proteinase inhibitor) homologs encoded by poxvirus, the influenza virus protein NS1, the vaccinia virus protein B15N or effectors molecules secreted by *Pseudomonas aeruginosa* or *Yersinia* spp. (183). The necroptosis executor RIP3 can be sequestered by MLKL homologs produced by poxviruses (184). RIP1 involved in TLR3 and 4 signaling pathways (**Figure 4**) is also frequently found as a target. Virus or bacteria effectors can modulate the PTM of RIP1 such as phosphorylation or ubiquitination, thereby affecting its kinase activity and its cell death-promoting ability (apoptosis or necroptosis). Thus, the latent membrane protein 1 (LMP1) from Epstein-Barr virus (EBV) and op-IAP produced by baculovirus can promote the poly-ubiquitination of RIP1 (162, 185) and the *Yersinia pestis* effector YopJ/P modulates the phosphorylation status of RIP1 by targeting the kinases TAK1 and IKK or MK2 (186). Pathogens can bypass host defense mechanisms by blocking signaling pathways just downstream of the pathogen recognition-receptor. This is illustrated by the enterohemorrhagic bacteria type 3 that produces a protease that cleaves the RHIM domain owned by RIP1, RIP3, TRIF, the adaptor proteins involved in TLR3 and TRL4-mediated signaling pathways and the cytosolic DNA sensor DAI (187). On the other hand, RHIM-homotypic interaction that mediates the assembly of the necrosome, as well as the recruitment of RIPs to sensors (**Figure 4**) can be affected by the presence of viral RHIM-containing proteins such as the proteins ICP6 and ICP10 produced by Herpes simplex virus (188), vaccinia virus innate immune evasion protein E3 (189), or vIRA encoded by the murine cytomegalovirus (190).

What is the situation in plants? Biotrophic or hemibiotrophic phytopathogens have to keep plant cells alive to ensure their infection cycle. In this way, they secrete many effectors which target receptors or key signaling components to suppress host immunity triggered by their own invading patterns (191, 192). As examples, RipAY produced by *Ralstonia solanacearum* inhibits SA-dependent defense responses and HR induced by the effector RipE1 in *Nicotiana benthamiana* (193), and RipAK suppresses catalase activity and HR of *Nicotiana tabacum* (194). *Phytophtora infestans* AVR3a targets the E3 ligase CMPG1 and suppresses HR induced by the elicitin INF1 in *Nicotiana benthamiana* (195, 196). In addition, it has been shown that *P. infestans* PexRD2 interacts with the KD of MAPKKKϵ, a positive regulator of cell death, increasing the susceptibility of *Nicotiana benthamiana* to this pathogen (197).

Inversely, necrotrophic phytopathogens favor plant cell death to ensure the infection spreading. For example, the broad host range necrotrophic plant pathogen *Sclerotinia sclerotiorum* secretes oxalic acid (OA) which is considered as a key molecule for its pathogenesis. It has been shown that OA has opposite roles: i) to suppress host oxidative burst and then HR at early state of infection allowing the establishment of the pathogen and ii) to activate plant cell death, *via* ROS production, facilitating disease development (198, 199). Besides OA secretion, *Sclerotinia sclerotiorum* produces several effectors or toxins inducing plant cell death (200). For some of them, secreted in apoplast such as SsNE1-SsNE5, the death-inducing signal is mediated by the BAK1/SOBIR1 receptor complex (200). The involvement of these RLK was already highlighted for the necrotizing activity of the xylanase BcXYG1 secreted by *Botrytis cinerea* (201). Others would be internalized in host cells as the “effector-like” protein SsSSVP1 which interacts with QCR8, a subunit of plant cytochrome complex in mitochondrial respiratory chain (202). This leads to the loss of function of QCR8 and to plant cell death induction. Actually, numerous phytopathogens secrete several toxins or effectors to induce cell death. Around 180 apoplastic cell death-inducing proteins (CDIPs) have been identified and for some of them, the associated receptors are known (203, 204). Other toxins are internalized in host cells and interact in some case with NB-LRR (182). Thus, the Arabidopsis susceptibility to *Cochliobolus victoriae* is due to the interaction between the secreted toxin victorin with the NB-LRR LOV1. In this case, the pathogen co-opts HR to facilitate its development (205).

A beneficial effect of cell death for pathogens to ensure infection cycle has also been described in mammals. It is well illustrated by the human immunodeficiency virus (HIV) that hijacks the immune surveillance by promoting the pyroptosis of immune cells (CD4^+^ lymphocytes) (206).

## Concluding Remarks

Although number of questions still remain to address, the intense research of the scientific communities on innate immunity in mammals during the three last decades and recent technological advances gave rise to a relative clear scheme of the cell death-signaling pathways activated in response to immune receptors (**Table 1**). By comparison, the understanding of the immune receptor-induced cell death signaling pathways remains incipient in plants although the use of Arabidopsis mutants allowed the identification of signaling molecules and regulators of HR (207–209). Up to date, more than 600 RLKs and 100 NLRs have been inventoried but only few have been characterized and many have not even been identified yet. The abundance of immune receptors, the different processes used for their activation as well as the diversity of cellular models make the decoding of cell death signaling pathways very difficult. The generic name HR does not reflect the complexity of signaling pathways and, as in mammals, recognition of PAMPs, DAMPs or effectors does probably not lead to the engagement of one unique cell death response but likely activates different cell death-signaling pathways with specific features and outcomes. The cell death signal also likely depends on the cell type and plant species.

**Table 1:** Main signaling pathways driving cell death or transcriptional reprogramming in response to activation of membrane-associated or intracellular immune receptors in animals and plants.

		Animals		Plants
	**Receptors**	** TLRs, CLRs**		**RLKs, RLPs**
**Membrane-associated receptors**	Signal transduction domain	homotypic interacting domains		kinase domains
	Mechanisms of signal transduction	assembly of multiprotein complexes in which executors are activated by proximity		PRR oligomerization, kinase activation & trans-phosphorylation
	Signaling pathways leading to transcriptional reprogramming	MAPK, NF-κB or IRFs		MAPKs and phosphorylation-dependent kinases ROS/NO production Ca^2+^ influx transcription factors activation Phyto-cytokines secretion
		(MyD88, TRIF or RIP1-dependent)		
	**Cell Death**	**Necroptosis**	**Apoptosis**	Hypersensitive Response (HR)*
	Cell death signaling, executors, associated features, regulation	Signaling platforms:		
		Necrosome	Ripoptosome	Ions fluxes across the PM
				Production of NO and ROS
		Key executors:		Inhibition/degradation of cell death repressors (HD2s…)
		RIP3,	RIP1,	
		pore-forming MLKL	Caspases cascade	Proteases activation (metacaspases in the cytosol, VPEs in the vacuole)
		(RIP1 dependent	(caspase-8, -3, -7)	
		or independent)		Phyto-cytokines secretion
		associated features:		Chromatine condensation, nucleus disruption,
		ROS, membrane permeabilisation	Silent form of cell death	vacuolar collapse
**Intracellular receptors**	**Receptors**	** NLRs, RLRs, ALRs**		**NLRs**
	Signal transduction	Homotypic interacting domains		Proteins interaction
	Mechanisms of signaling activation	Assembly of multiprotein complexes in which executors are activiated by proximity		Effectors detection (direct) or proteins modification (indirect)
	Signaling pathways leading to transcriptional reprogramming	NODosome assembly leading to		Unknown
		MAPK & NF-κB		(direct activation of transcription factors by NLRs or linked to pore-forming structures in the PM?)
	**Cell Death**	** Main: Pyroptose**		**Hypersensitive response (HR)**
		** Alternative: Necroptose**		
	Signaling pathways/platforms leading to cell death	Inflammasome		Resistosome, other NLR complexes
	Key executors of cell death	Caspase 1,		Unknown (pore-forming Ca^2+^-dependent activation of proteolytic cascade?)
		pore-forming Gasdermin D		
	Associated-features, regulations	IL-1β, IL-18 release, membrane permeabilisation, ionic unbalance, ROS production		ROS & NO production, phytohormones accumulation, membranes permeabilization, release of active proteases and phyto-cytokines

ALR, AIM2 (absent in melanoma 2)-like receptors; AtEDS1, Arabidopsis thaliana enhanced disease susceptibility 1; AtNRG1, Arabidopsis thaliana N-requirement gene 1; AtSAG101, Arabidopsis thaliana senescence-associated gene101; CLR, C-type lectin receptors; HD2, Histone deacetylase; HR, Hypersensitive response; IL, Interleukin; IRF, Interferon-regulatory factors; MAPK, Mitogen-activated protein kinase; MLKL, Mixed lineage kinase domain-like; MyD88, myeloid differentiation factor 88; NLR, Nucleotide-binding and oligomerization domain (NOD)-Like Receptor [animals] or Nucleotide-Binding Domain (NBD)-containing LRRs [plants]; NO, Nitric oxide; PLCP, papain-like cysteine proteases; PM, plasma membrane; RLK, Receptor-Like Kinase; RIP, receptor-interacting kinase; RLP, Receptor-Like Protein (contains a short cytoplasmic domain devoid of kinase activity); RLR, RIG-I-like receptors; ROS, Reactive oxygen species; TLRs, Toll-like receptors; TRIF, TIR-domain-containing adaptor-inducing IFN-b; VPE, Vacuolar processing enzyme. *HR cell death induced by membrane-associated receptors is an exceptional outcome.

The plant immune response is associated with different biochemical modifications and cellular signals that include MAPK activation, oxidative and nitrosative bursts, calcium fluxes, phytohormones production, protease activation, and transcriptional reprogramming (5). Studies analyzing their involvement in immune receptor-induced cell death reported controversial results and their direct role in transducing cell death signal is still debated. A closer characterization of the spatial and temporal aspects of these cellular events could probably provide a better view of their involvement. By putting animal models into perspective, we can hypothesize that the stimulation of plant membrane PRRs or NLRs have the ability to engage (i) signaling pathways leading to transcriptional reprogramming responsible for phytohormones production and expression of defense genes, and (ii) a cell death signaling pathway that can culminate into ions imbalance and in the rupture of plasma membrane, both regulated by interplays and cross-regulation mechanisms.

The analysis of plant NLRs structure showed that the signal transduction domains belong to conserved homotypic interacting domain family. This suggests that mechanisms of activation involve the assembly of multiprotein platforms. A range of evidences indeed suggests that plant NLRs form signaling platforms to promote cell death (39, 96–99, 204, 210), as observed for mammals NLRs. The recent works of Wang and colleagues (96, 210) highlighting the presence of a resistosome that can translocate into plasma membrane to probably form pore-like structures provided very important elements into understanding the plant NLR-mediated cell death signaling. It is interesting to note that the lumen of the funnel-shape structure found in the resistosome has negative electrostatic potentials given by two negatively charged glutamic acid residues (96). Such negatively charged glutamate residue is thought to be critical for anion selectivity in several human Ca^2+^ voltage-dependent channels (211, 212). It is thus tempting to speculate that this selectivity for cations could be associated with a death process. The similarities between the ZAR1-resistosome and NLR inflammasomes as discussed in the recent review of Xiong et al. (213) suggest that the NLR-mediated cell death signaling pathway could be a conserved process.

While the role and mechanisms of activation of proteases (i.e., caspases-1, -8, and -3) in PRR-induced signaling pathways start to be well characterized in mammals, the activation mechanisms of plant proteases and their importance in the immune-receptor-induced HR remain important questions to address. Indeed, our knowledge about the proteolytic cascades involved in HR cell death is really scarce and fragmentary. Of interest, AtMC4 has been recently shown to be activated by Ca^2+^ (136), suggesting a link between calcium influx, metacaspase activation and release of mature phytocytokines. However, many things remain to discover such as the different proteolytic cascades involved, their initiation and regulation during plant HR.

## Future Applications for Plant Protection

In Arabidopsis, the screening of vast mutant collections and naturally occurring ecotypes, as well as forward genetic approaches, has led to the successful identification of novel immune receptors involved in HR cell death. In crops, analyzing genomic variations within different cultivars but also the “wild” relative species and their introgression lines allowed to map the Quantitative Trait Loci (QTLs) related to disease resistance. Although QTLs will mostly carry *R*-genes, they may also contain PRR genes (encoding RLKs or RLPs). In a scientific point of view, it is interesting to note that PRRs can be successfully transferred from one plant species to another to provide a novel source of resistance. A very effective demonstration was achieved in tomato (from the Solanaceae family), where the transfer of the EFR RLK receptor (from the Brassicaceae family) led to a great resistance of plants against a wide range of different bacterial pathogens (214). Different studies also showed that the ectodomain and KDs from distinct PRRs can be combined in order to form chimera receptors with preserved signal transduction. Such a chimeric receptor was built from the chitin-binding ectodomain of OsCEBiP and the KD of Xa21. This chimera receptor was able to initiate HR in rice thus conferring to the plant a highly improved resistance to the fungus *Magnaporthe oryzae*. This strategy thus represents an interest for practical use in disease resistance engineering (215).

Analysis of the polymorphism occurring in plant immune receptors or cell death regulators in different cultivars or species could lead to the identification of more efficient variants. As an alternative to the transgenic approach, conventional breeding can be assisted by the use of molecular markers that help to deliver the desired gene into the crop, pyramiding it with other genes important for the plant resistance such as *R*-genes.

All over, the immune receptor-based breeding, the transfer and creation of novel chimeric PRRs might be applicable as an alternative in agriculture disease and pest management, as a “tailored immune-receptor therapy” that might provide more durable and broader resistance when associated with *R*-genes.

## Author Contributions

TR, M-CH, DW, AZ, LD, and BP contributed equally to the writing of this review. All authors contributed to the article and approved the submitted version.

## Funding

TR, M-CH, and BP are financially supported by Agence Nationale de la Recherche (ANR) and Agence Française pour la Biodiversité (AFB) (“ChitoProtect” project, grant # ANR-19-ECOM-0008) and Institut Carnot Plant2Pro (“VitiLYKs” project, grant # C4520). TR, M-CH, DW, AZ, LD, and BP have been supported by the Ministère de l’Enseignement Supérieur, de la Recherche et de l’Innovation (MESRI). DW is supported by the MESRI, Investissements d’Avenir program, project “Initiatives Science Innovation Territoire Economie en Bourgogne-Franche-Comté” (“Structure, Function and Roles of Nitric Oxide Synthases in Algal Responses to Environemental Stresses (NOISELESS)” project, grant # RA18041.AEC.IS) and the ANR (“Algae-Nitric Oxide Synthases” project, grant # ANR-18-CE20-0022-02). LD is supported by grants from “La Ligue contre le cancer” Comité de la Côte d’Or, the “Conseil Regional de Bourgogne-Franche-Comté”, the ANR, (“Investissements d’Avenir” program, grant # ANR-11-LABX-0021) and the European Union program FEDER.

## Conflict of Interest

The authors declare that the research was conducted in the absence of any commercial or financial relationships that could be construed as a potential conflict of interest.
